# Copper homeostasis-associated gene PRNP regulates ferroptosis and immune infiltration in breast cancer

**DOI:** 10.1371/journal.pone.0288091

**Published:** 2023-08-03

**Authors:** Changwei Lin, Jiaqing He, Xiaopei Tong, Liying Song

**Affiliations:** 1 Department of Gastrointestinal Surgery, The Third Xiangya Hospital of Central South University, Changsha, Hunan. P. R. China; 2 Xiangya School of Pharmaceutical Sciences, Central South University, Changsha, Hunan, P.R. China; 3 Department of Pharmacy, The Third Xiangya Hospital, Central South University, Changsha, Hunan, P.R. China; The First Affiliated Hospital of Nanjing Medical University, CHINA

## Abstract

Breast cancer (BRCA) is one of the most common cancers in women. Copper (Cu) is an essential trace element implicated in many physiological processes and human diseases, including BRCA. In this study, we performed bioinformatics analysis and experiments to determine differentially expressed copper homeostasis-associated genes in BRCA. Based on two Gene Expression Omnibus (GEO) datasets, the copper homeostasis-associated gene, prion protein (PRNP), a highly conserved ubiquitous glycoprotein, was significantly down-regulated in BRCA compared to normal tissues. Moreover, PRNP expression predicted a better prognosis in BRCA patients. Kyoto Encyclopedia of Genes and Genomes (KEGG) pathway analysis indicated that PRNP was potentially linked with several cancer-associated signaling pathways, including regulation of inflammatory response and oxidative phosphorylation. To validate the biological functions of PRNP, we overexpressed PRNP in BRCA cell lines, MDA-MB-231 and BT-549. CCK8 assay showed that PRNP overexpression significantly increased the sensitivity of gefitinib in BRCA cells. Overexpression of PRNP resulted in increased reactive oxygen species (ROS) production upon gefitinib treatment and ferroptosis selective inhibitor, ferrostatin-1 attenuated the enhanced ROS production effect of PRNP in BRCA cells. PRNP expression was positively correlated with macrophages, Th1 cells, neutrophils, and B cells, while negatively correlated with NK CD56 bright cells and Th17 cells in BRCA. Single-cell analysis showed that PRNP was highly expressed in M1 phenotype macrophages, essential tumor-suppressing cells in the tumor stroma. Therefore, our findings suggest that PRNP may participate in ROS-mediated ferroptosis and is a potential novel therapeutic target of chemotherapy and immunotherapy in BRCA.

## Introduction

Breast cancer (BRCA) is one of the most frequently diagnosed cancers worldwide with an estimated 2.3 million new cases (11.7%) in 2020, thus surpassing lung cancer [1, 2]. BRCA is considered a heterogeneous disease involving genetic and environmental factors. The prognosis of breast cancer depends on the stage at which it is diagnosed and its biological characteristics [3]. Triple-negative breast cancer (TNBC) accounts for 10–15% of all breast cancer cases and is characterized by high invasiveness and metastatic potential with poor patient prognosis and insensitivity to endocrine therapy or HER2 treatment, and is prone to relapse [4, 5]. Standardized BRCA treatment regimens are lacking. Existing research has identified that bevacizumab combined with chemotherapeutics can be used to treat TNBC with no significant increase in the survival time [6]. Selective epidermal growth factor receptor (EGFR) tyrosine kinase inhibitors such as gefitinib are ineffective in treating TNBC patients and novel approaches such as the combination of raloxifene have been approved and can promote the effect of gefitinib in TNBC [7]. However, the discovery of novel prognostic biomarkers to benefit targeted therapies is an urgent challenge in clinical settings.

Cu is an essential micronutrient for maintaining cellular homeostasis. Its levels must be tightly regulated, as excessive intracellular Cu can lead to oxidative stress, ultimately disrupting cellular function [8]. Cu homeostasis is involved in cell proliferation, angiogenesis, and metastasis. Several reports have demonstrated that Cu homeostasis and Cu binding proteins are involved in many cancers, including BRCA, colorectal cancer, and lung cancer [9, 10]. For example, downregulated Cu homeostasis-related gene, FOXO1, is a novel indicator for the prognosis and immune response of BRCA [11]. Previous research suggests that COMMD3 controls Cu levels and acts as a negative regulator by modulating Cu homeostasis in cancer cells. The loss of COMMD3 promotes aggressive behavior in BRCA, suggesting the potential use of Cu chelation in COMMD3 low-expressing cells to improve disease progression and metastasis of breast cancer [12]. However, the specific effects and potential mechanisms of Cu homeostasis-associated genes in BRCA pathogenesis and therapeutic response are not yet fully understood.

The prion protein-encoding gene (PRNP) encodes the major prion protein (PrP). Prion disease is associated with the expression of PrP, which is abundant in the nervous system and participates in neuronal growth and survival [13]. In cancer cells, the role of PrP is controversial in cell proliferation, invasion, metastasis, and treatment resistance [14–17]. In head and neck squamous cell carcinoma, PrP is significantly correlated with lymph node metastasis progression and worse prognosis and acts through the upregulation of HSPA4, HSP90AA1, and HIF1A [18]. However, Angelita *et al*. found that PrP ablation induced aggressiveness and embolization by regulating cell adhesion and differentiation [19]. In breast cancer, PrP exhibits an antiapoptotic effect on the cytotoxic action of tumor necrosis factor α (TNF) and contributes to TNF-resistance [20]. Thus, for a better understanding of the regulation of PRNP, we employed multiple bioinformatic platforms and performed experiments to investigate the potential mechanism of PRNP in BRCA. In this study, two Gene Expression Omnibus (GEO) datasets were utilized to select differentially expressed genes (DEGs) that influence the progression and prognosis of BRCA. We found that the copper homeostasis-related gene, PRNP, was aberrantly expressed and correlated with a better prognosis. Enrichment and immune infiltration analyses were used to illustrate the potential signaling pathways and biological functions of PRNP. Moreover, PRNP was found to be associated with cell viability and can regulate ferroptosis following gefitinib treatment in BRCA cells, suggesting the novel function of PRNP in targeted diagnosis and treatment of patients with BRCA.

## Materials and methods

### Data collection

Using the GEO database, we obtained two breast cancer-related datasets, including GSE21422 [21] and GSE31192 [22]. Based on the screening criteria of p < 0.05 and |logFC| > 0.75, DEGs were extracted between BRCA and normal groups. Cu homeostasis-associated genes were extracted from the MalaCards database (https://www.malacards.org/). The extracted DEGs were also subjected to intersection analysis with the set of copper homeostasis genes using Venn diagrams, and the co-differentially expressed genes (co-DEGs) were obtained.

### Gene expression and prognostic analysis

The GEO2R tool was used to analyze the level of PRNP expression in the tumor and normal control groups of the two GEO datasets. We performed an immunohistochemistry analysis for PRNP expression in breast tissues using the HPA database [23], and queried the clinical data of PRNP in breast cancer using The Cancer Genome Atlas (TCGA) database. The TNM plot [24] and the GEPIA2 database [25] were used to confirm the expression levels of PRNP in BRCA. The expression of co-DEGs and the patients’ prognoses were assessed using the Kaplan-Meier plotter [26] in the three BRCA datasets (GSE45255, GSE3494, and GSE1456). The overall survival (OS) was defined as the end point, and the hazard ratio (HR) was used to estimate the treatment effect of each gene.

### Correlation and enrichment analysis

The STRING database [27] was employed to construct a protein-protein interaction network for PRNP, as well as draw the correlation scatter plots of co-expressed genes using GEPIA2. Tumor Immune Estimation Resource 2.0 (TIMER2.0) [28] was utilized to generate correlation heat maps, while Linked-Omics [29] facilitated the derivation of gene ontology (GO) annotations and Kyoto Encyclopedia of Genes and Genomes (KEGG) pathways related to PRNP.

### Immune infiltration analysis

To assess the expression of PRNP in breast cancer tissues and the abundance of 24 tumor-infiltrating immune cells (TIICs), we employed the single sample gene set enrichment analysis (ssGSEA) [30]. The TIMER2.0 database was used for further validation for facilitating the assessment of TIIC abundance. We used the TISIDB database [31] to validate these immunological findings. The tumor microenvironment (TME) was further analyzed using the scRNA-seq database Tumor Immune Single-cell Hub 2 (TISCH2) [32].

### Measurement of reactive oxygen species (ROS)

We used the peroxide-sensitive fluorescent probe DCFDA/H2DCFDA (ab113851, Sigma) to detect cellular ROS levels. BT-549 and MDA-MB-231 cells were transfected with PRNP overexpression plasmids or control vectors using the Lipofectamine™ 3000 Transfection Reagent (L3000015, Invitrogen) for 24 hours. Then the cells were inoculated in six-well plates and treated with or without 0.1 μM Gefitinib for additional 48 hours. The ROS levels of each sample were determined by CytoFLEX Research Flow Cytometry (Beckman Coulter) at 488 nm excitation and 525 nm emission according to standard protocols.

### CCK-8 assay

BT-549 and MDA-MB-231 cells were seeded in 96-well plates (2×10^3^ cells/well) after transfection with PRNP overexpression and control plasmids for 24 hours. Gefitinib (0.1μM) was added to the medium for 48 hours. On the day of analysis, CCK-8 assays were conducted to examine cell viability following the manufacturer’s protocols. Briefly, 10 μl CCK-8 test solution (B34302, Bimake) was added into each well and incubated for 1 h. The samples’ optical density (OD) was detected at 450 nm using a VICTOR X2 microplate reader (PerkinElmer, USA).

### Western blotting

Protein extraction was performed according to a previously described protocol [33]. Protein samples were separated by SDS-PAGE, and separated proteins were transferred onto polyvinylidene difluoride membrane, and hybridized with the antibodies specific to PRNP (12555-1-AP, Proteintech) and β-actin (8432, Santa Cruz). Protein bands were visualized in the Bio-Rad ChemiDoc XRS system (Berkeley) using the HRP substrate chemiluminescence reagent (Millipore, United States).

## Results

### Identification of DEGs

**Fig 1:**
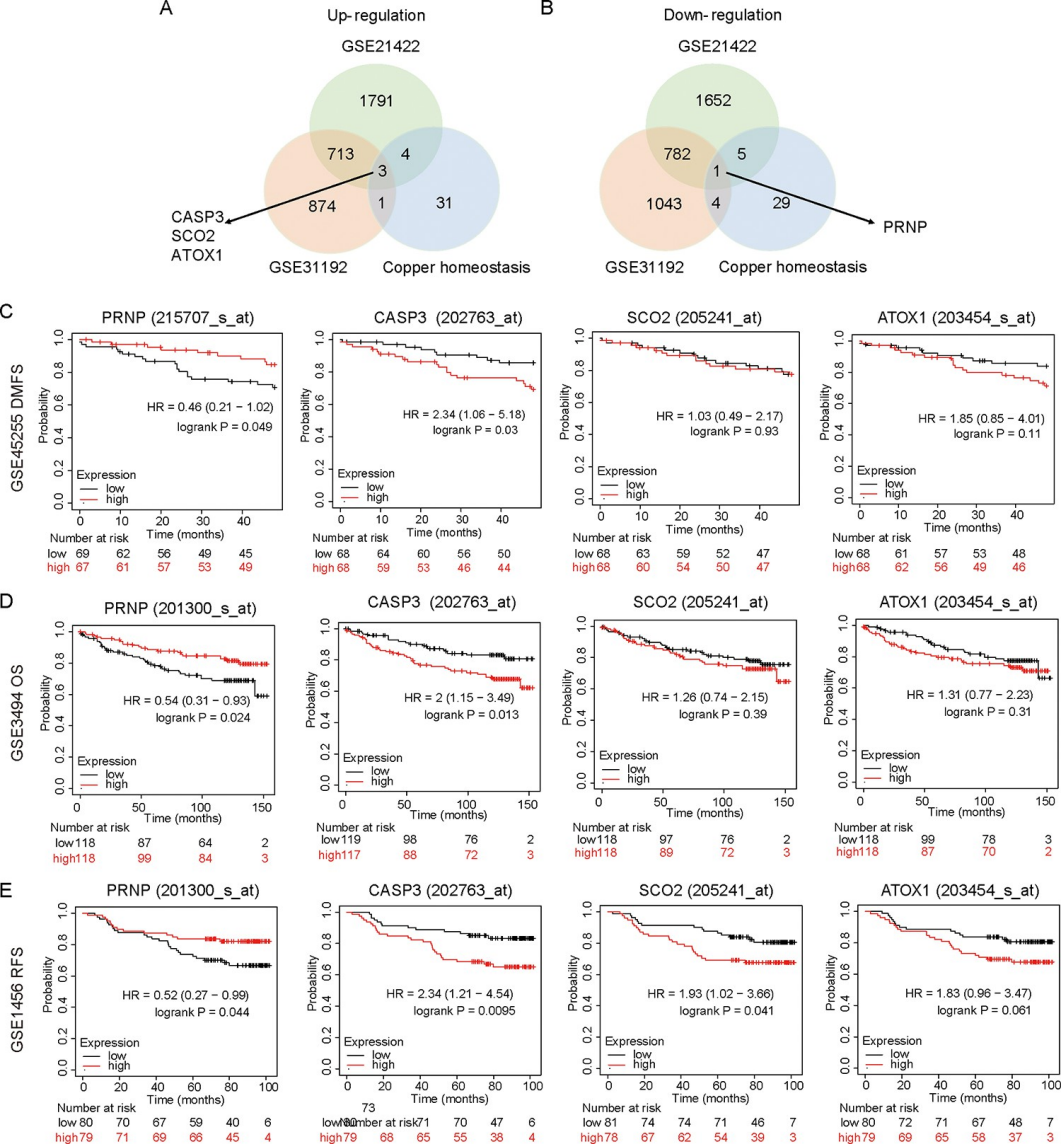
Co-differentially expressed copper homeostasis genes in two BRCA datasets. **(A)** Three upregulated copper homeostasis genes (CASP3, SCO2, ATOX1) in BRCA datasets. **(B)** A downregulated copper homeostasis gene (PRNP) in BRCA datasets. **(C-E)** Prognostic values of the levels of expression of PRNP, CASP3, ATOX1, and SCO2 in BRCA patients indicated by Kaplan-Meier plotter database analysis.

Based on screening criteria (p<0.05 and | log FC| >0.75), we analyzed the GSE21422 and GSE31192 datasets in the GEO database to identify DEGs between the normal and BRCA groups. There were 2511 up-regulated genes and 2440 down-regulated genes in GSE21422, and 1591 up-regulated genes and 1830 down-regulated genes in GSE31192. Cancers are thought to be linked with copper regulation, and tumorigenesis is significantly influenced by copper homeostasis. Using the Venn diagram, three up-regulated genes CASP3, SCO2, ATOX1, and a down-regulated gene, PRNP, were identified in the two GEO data sets associated with copper homeostasis (Fig 1A and 1B). The aberrant expression of PRNP in pan-cancer is shown in S1 Fig.

Nest, by using Kaplan-Meier curves, we examined the prognostic value of CASP3, SCO2, ATOX1, and PRNP in three GEO datasets, including GSE45255, GSE3494, and GSE1456. Fig 1C–1E show that patients with breast cancer with high PRNP expression had significantly favorable DMFS, OS and RFS, however, no consistent significance was observed based on the correlation of CASP3, SCO2, or ATOX expression with prognosis. These findings collectively indicated the promising roles of downregulated PRNP expression in BRCA patients.

### Expression and clinical significance of PRNP expression

**Fig 2:**
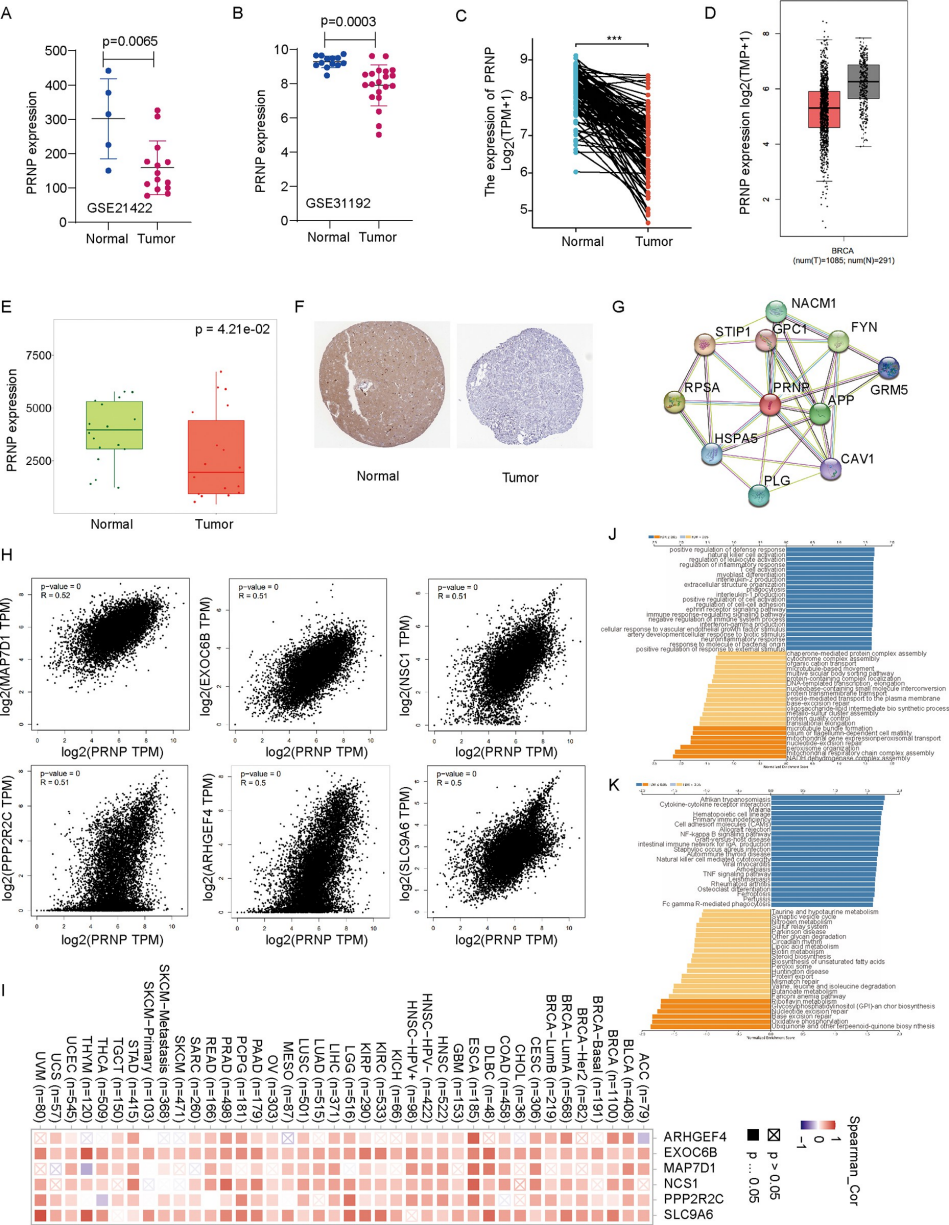
PRNP expression in breast cancer tissues. Compared with normal breast tissue, a significantly reduced PRNP expression was found in **(A)** GSE21422, **(B)** GSE31192, **(C)** TCGA-BRCA, and **(D)** GEPTA2. **(E)** The verification of down-regulated expression of PRNP using the TNM-plot database. **(F)** Immunohistochemical analysis indicates downregulated PRNP levels in BRCA patients. **(G)** Co-expression network and enrichment pathway analysis of PRNP. Ten experimentally identified PRNP-binding molecules were acquired using STRING. **(H)** The top six PRNP-correlated genes were analyzed by GEPIA2, including MAP7D1, EXOC66, NCS1, PPP2R2C, ARHGEF4, and SLC9A6. **(I)** Heatmap representation of the prognostic relevance of the top six PRNP-correlated genes in pan-cancer. **(J-K)** Bar chart of GO and KEGG enrichment analysis results for PRNP in BRCA.

In GSE21422 and GSE31192 datasets, we compared the expression of PRNP in breast cancer tissues to that in healthy tissues and found that the expression of PRNP was lower in BRCA tissues (Fig 2A and 2B). Next, information from TCGA-BRCA revealed that breast cancer had considerably lower PRNP expression (Fig 2C). Additionally, the GEPIA2 platform revealed decreased expression of PRNP in BRCA patients (p<0.05) (Fig 2D). The TNM plot also confirmed the decrease in PRNP expression in the BRCA group based on the gene microarray data (p = 4.21e-02) (Fig 2E). Using HPA datasets, the immunohistochemical assay showed that PRNP was downregulated in the BRCA group (Fig 2F), further confirming the above results. Additionally, using clinical data from TCGA database, we examine the link between PRNP expression and clinical features. The BRCA patients were divided into two groups (PRNP high or PRNP low) based on PRNP expression. As shown in Table 1, low PRNP expression is more common in luminal B patients; in contrast, basal-like patients tend to have higher PRNP expression. These findings might present a fresh viewpoint on PRNP as a novel BRCA biomarker.

**Table 1:** Association of clinical characteristics and PRNP expression in BRCA patients.

Characteristics	Low expression of PRNP	High expression of PRNP	p-value
n	543	544	
Pathologic T stage, n (%)			0.790
T1	133 (12.3%)	145 (13.4%)	
T2	317 (29.2%)	314 (29%)	
T3	73 (6.7%)	67 (6.2%)	
T4	19 (1.8%)	16 (1.5%)	
Pathologic N stage, n (%)			0.974
N0	256 (24%)	260 (24.3%)	
N1	179 (16.8%)	180 (16.9%)	
N2	55 (5.1%)	61 (5.7%)	
N3	38 (3.6%)	39 (3.7%)	
Pathologic M stage, n (%)			0.934
M0	444 (48%)	461 (49.8%)	
M1	10 (1.1%)	10 (1.1%)	
PR status, n (%)			< 0.001
Negative	130 (12.5%)	212 (20.4%)	
Indeterminate	2 (0.2%)	2 (0.2%)	
Positive	387 (37.3%)	305 (29.4%)	
ER status, n (%)			< 0.001
Negative	69 (6.6%)	171 (16.5%)	
Indeterminate	2 (0.2%)	0 (0%)	
Positive	448 (43.1%)	349 (33.6%)	
HER2 status, n (%)			< 0.001
Negative	243 (33.3%)	317 (43.5%)	
Indeterminate	9 (1.2%)	3 (0.4%)	
Positive	96 (13.2%)	61 (8.4%)	
PAM50, n (%)			< 0.001
Normal	4 (0.4%)	36 (3.3%)	
LumA	303 (27.9%)	261 (24%)	
LumB	149 (13.7%)	57 (5.2%)	
Her2	51 (4.7%)	31 (2.9%)	
Basal	36 (3.3%)	159 (14.6%)	
Menopause status, n (%)			< 0.001
Pre	92 (9.4%)	138 (14.1%)	
Peri	17 (1.7%)	23 (2.4%)	
Post	381 (39%)	325 (33.3%)	

### Co-expression analysis for PRNP

Using the STRING database, we investigated the co-expressed genes of PRNP in BRCA patients. The top ten experimentally identified PRNP-binding molecules were acquired as shown in Fig 2G. The top six PRNP-correlated genes were analyzed by GEPIA2, including MAP7D1, EXOC66, NCS1, PPP2R2C, ARHGEF4, and SLC9A6 (Fig 2H). TIMER 2.0 was used to explore the prognostic relevance of the six genes having strong correlation coefficients in pan-cancer, and the results are displayed as a heat map (Fig 2I). PRNP showed a positive correlation with the six aforementioned genes in various cancer types (p<0.05). We used Linked-Omic to perform KEGG pathway and GO enrichment analyses for PRNP. Co-expressed gees were primarily involved in certain immunization processes, such as positive regulation of defense response, natural killer cell activation, regulation of leukocyte activation, and regulation of inflammatory response (Fig 2J). Additionally, the top four enriched pathways were identified in KEGG enrichment analysis, including ubiquinone and other terpenoid-quinone production, oxidative phosphorylation, base excision repair, and nucleotide excision repair (Fig 2K).

### PRNP regulates ferroptosis and sensitizes BRCA cells to gefitinib

**Fig 3:**
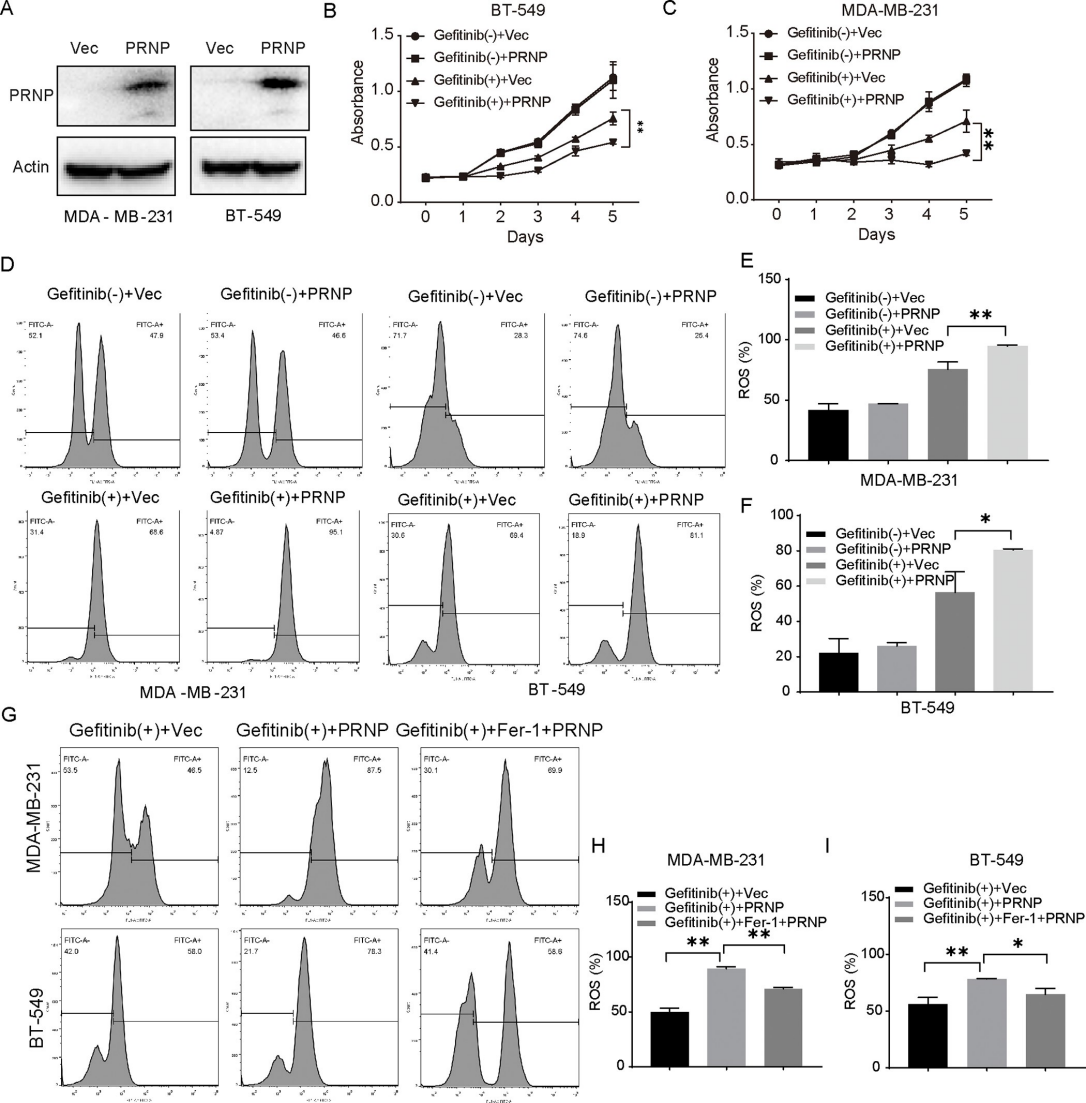
PRNP overexpression sensitizes BRCA cells to gefitinib and improves cellular ROS levels. **(A)** BT-549 and MDA-MB-231 cells were transiently transfected with Flag-PRNP. PRNP protein expression was measured by western blotting. **(B–C)** CCK8 experiments were performed and overexpression of PRNP was found to increase the inhibitory effect of gefitinib and the sensitivity of BRCA cells to gefitinib. **(D–F)** Overexpression of PRNP resulted in increased ROS production after gefitinib treatment. **(G-I)** Ferroptosis selective inhibitor ferrostatin-1 attenuated enhanced ROS production due to PRNP overexpression. (*p < 0.05, **p < 0.01).

BT-549 and MDA-MB-231 cells were transfected with a PRNP overexpression plasmid and validated by western blotting (Fig 3A). Through the CCK8 experiment, overexpression of PRNP sensitized BRCA cells to gefitinib was validated (Fig 3B and 3C). Recent studies have shown that induction of ferroptosis promotes gefitinib sensitivity in BRCA through ROS production [34]. Overexpression of PRNP resulted in increased ROS production after gefitinib treatment (Fig 3D to 3F). Treatment with the ferroptosis inhibitor, ferrostatin-1, significantly attenuated enhanced ROS production due to PRNP overexpression (Fig 3G and 3I). These results suggested PRNP may participate in gefitinib sensitivity in BRCA cells by regulating ferroptosis.

### Role of PRNP in immune regulation

**Fig 4:**
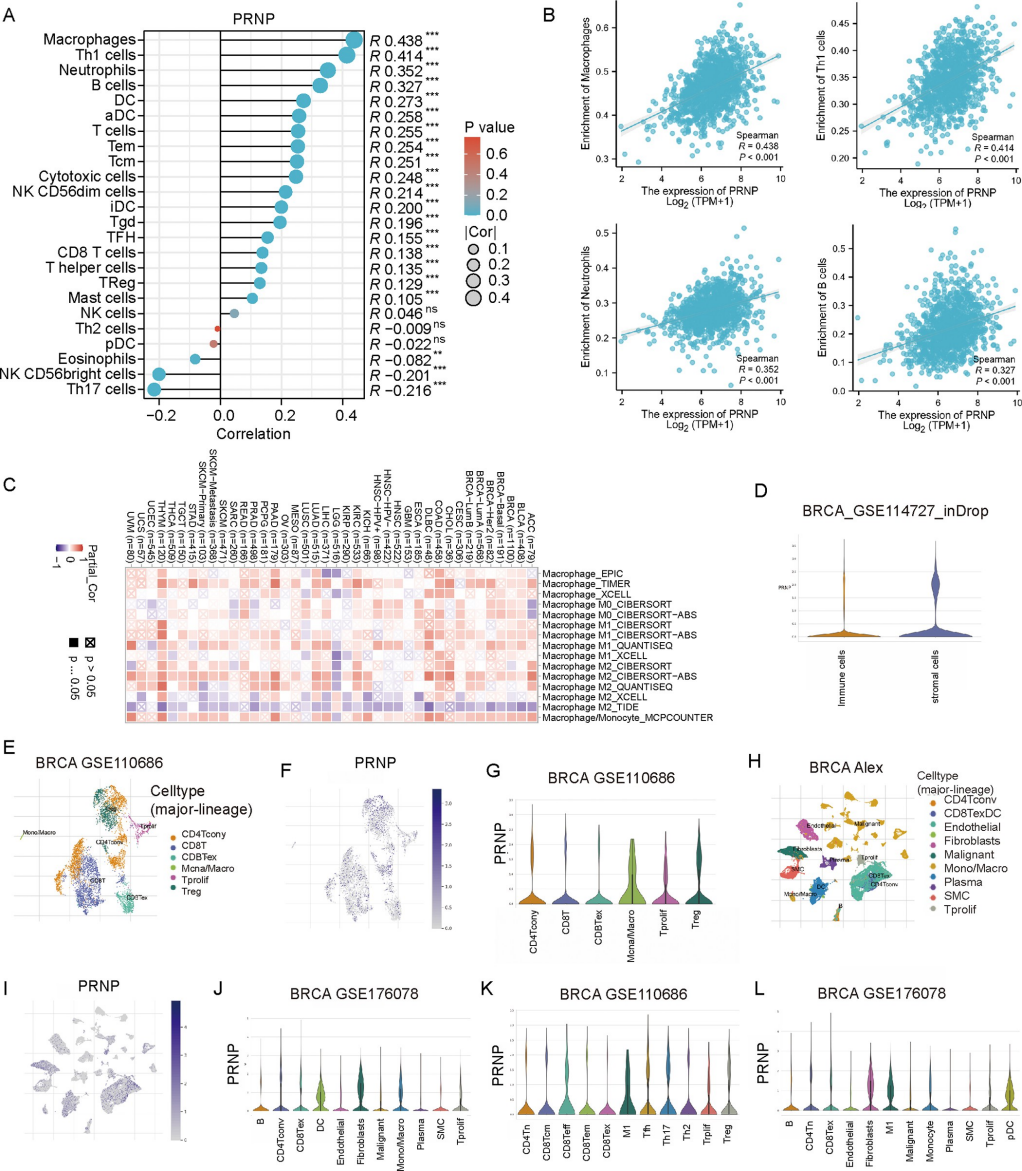
Relationship between PRNP expression and immune infiltrating cells in BRCA. **(A)** Twenty-four types of immune-infiltrating cells related to PRNP expression. The absolute values of Spearman R-value are indicated by the size of round dots. **(B)** Scatter plots show the correlation between PRNP expression and macrophages, Th1cells, neutrophils, and B cells. **(C)** The heat map was drawn based on TIMER2.0 data to further evaluate the pan-cancer data for macrophages and PRNP expression was found to be correlated with macrophages M0 and M1. **(D)** PRNP expression was positively linked with stromal cells in BRCA. Single-cell sequencing analysis for PRNA expression in BRCA. PRNP was positively related to macrophages **(E-J)** and M1 macrophages **(K-L)**.

Based on statistics obtained from TCGA database, we assessed the role of PRNP expression in immune regulation in BRCA. According to the findings, PRNP expression was positively associated with macrophages, Th1 cells, neutrophils, and B cells, while negatively correlated with NK CD56 bright cells and Th17 cells (p<0.001) (Fig 4A). The positive correlations between PRNP expression and macrophages, Th1 cells, neutrophils, and B cells are shown in Fig 4B. The TIMER2.0 database was utilized to further evaluate the pan-cancer data for the expression of PRNP and macrophages. As shown in Fig 4C, PRNP correlated positively with M0 and M1 macrophages. Through the analysis of single-cell data in TISCH database, PRNP was notably expressed in stromal cells of BRCA_GSE114727_inDrop (Fig 4D). Based on BRCA_GSE110686, BRCA_Alex, and BRCA_GSE176078, the major lineage analysis showed PRNP was mainly expressed in macrophages (Fig 4E to 4J), especially M1-like macrophages (Fig 4K and 4L).

**Fig 5:**
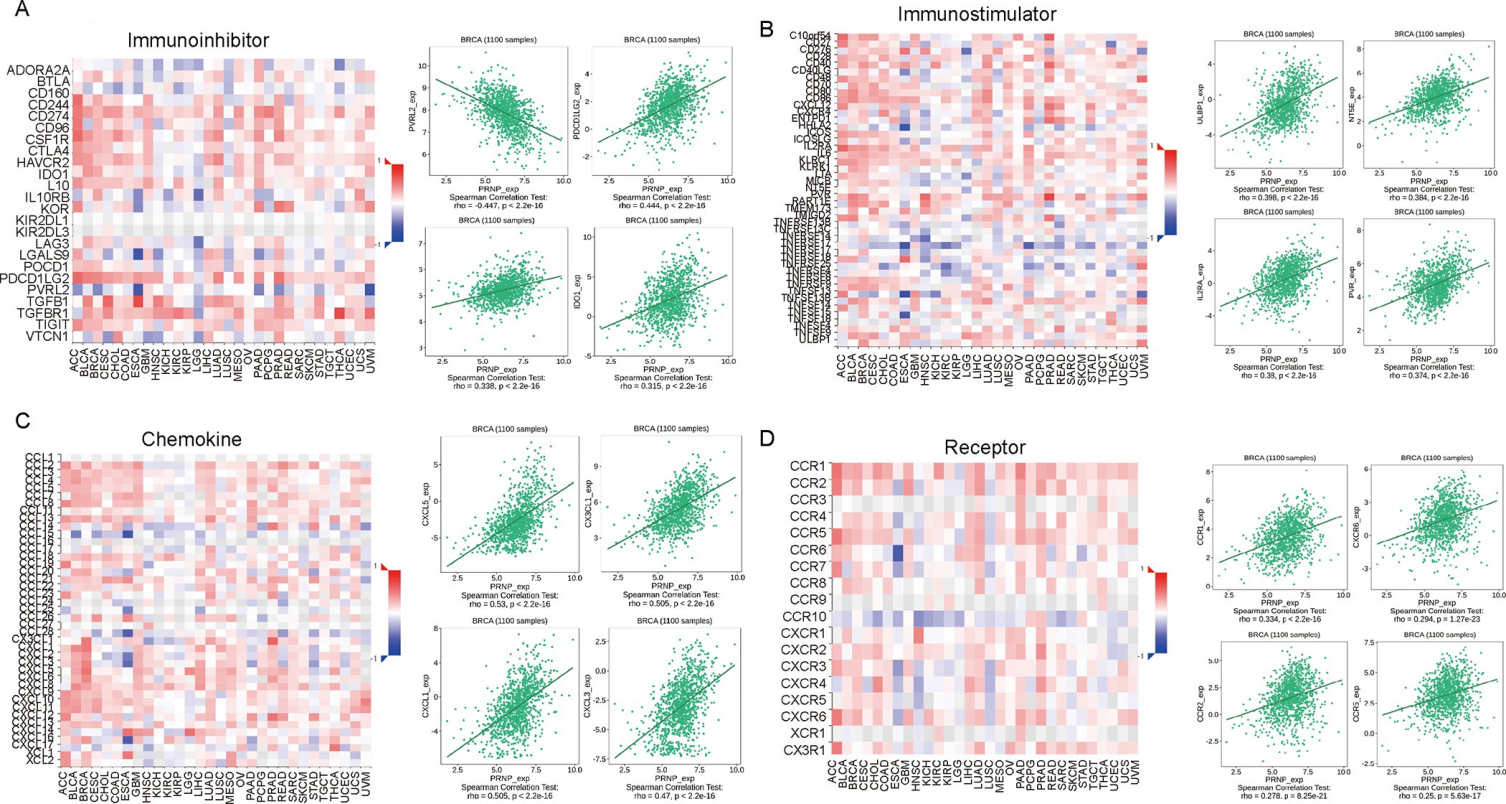
Associations between PRNP expression and immune inhibitors, immunomodulators, chemokines, and receptors from the TISIDB database. **(A)** The top four immune inhibitors linked with PRNP expression. PVRL2 was negatively correlated with PRNP levels, while those of PDCD1LG2, IL10RB, and IDO1 were positively correlated. **(B)** The top four immune-stimulators including ULBP1, NT5E, IL2RA, and PVR correlated positively with PRNP expression. **(C)** The top four chemokines, including CXCL5, CX3CL1, CXCL1, and CXCL3, correlated positively with PRNP expression. **(D)** The top four receptors, including CCR1, CXCR6, CCR2, and CCR5, correlated positively with PRNP.

Based on the TISIDB database, we also investigated the role of PRNP expression in immune-related characteristics of BRCA, including immune inhibitor, immune stimulator, chemokines, and their receptors. The top four immune inhibitors (PVRL2, PDCD1LG2, IL10RB, and IDO1) and top four immune stimulators (ULBP1, NT5E, IL2RA, and PVR), respectively, are shown in Fig 5A and 5B. The top four chemokines (CXCL5, CX3CL1, CXCL1, and CXCL3) and four receptors (CCR1, CXCR6, CCR2, and CCR5) are shown in Fig 5C and 5D. These results implied that abnormal PRNP expression can control the immunological microenvironment in BRCA patients.

## Discussion

In this study, we explored the association of Cu homeostasis genes with the progression and prognosis of BRCA patients. From the GEO database, we initially selected two BRCA datasets and screened co-DEGs among the two BRCA datasets and the Cu homeostasis gene dataset. We found that PRNP was significantly down-regulated in BRCA tissues, and the high expression of PRNP was related to a better prognosis in BRCA patients. Additionally, we examined the co-expressed genes of PRNP and discovered the potential function of PRNP. In BRCA cells, PRNP was associated with cell viability inhibition and increasing ROS levels following gefitinib treatment. Moreover, the ROS induction effect of PRNP could be reversed by treatment of a ferroptosis inhibitor, ferrostatin-1. These results indicated that PRNP might affect the prognosis and treatment of breast cancer by regulating ferroptosis.

Cell death is a process of cell suicide to maintain human health, which is fundamentally vital in novel target identification for cancer treatment. Mainly, cell death types include necroptosis, pyroptosis, autophagy, apoptosis, ferroptosis, and cuproptosis [35, 36]. Recently, studies have shown that abnormal Cu levels are a key marker of numerous diseases in the human body. Cu ions control crucial tumor characteristics, such as unrestricted proliferation, angiogenesis, metastasis, immune cell infiltration, and immunological escape [37]. The PrP protein plays a role in stabilizing cellular Cu homeostasis under oxidative conditions. It is also implicated in the occurrence of gastric cancer, prostate cancer, and glioblastoma [38]. In our study, PRNP was down-regulated and was associated with a good prognosis in BRCA. Overexpression of PRNP attenuated the gefitinib sensitivity by up-regulating ROS levels and ferroptosis. Ferroptosis is a cell death type that induces defense processes against cancer naturally by interfering with normal fatty acid metabolism [39]. Existing research indicates that ferroptosis participates in the pathogenesis of various diseases including neurodegenerative diseases [40], auto-immune diseases [41], and various cancers, including BRCA [42]. Notably, the interconnection of ferroptosis and cuproptosis occurs in many diseases ranging from intracellular infection to cancer. Furthermore, the balance between ROS and antioxidants plays an essential role both in triggering ferroptosis and cuproptosis [43]. The co-regulators of cuproptosis and ferroptosis to identify patients eligible for chemotherapeutic drugs sensitivity may become a critical factor in the diagnosis and prognosis of BRCA.

The clinical prognosis of cancer patients is highly correlated with the TME [44]. Immune cell infiltration is a distinctive pattern of host immune response and is crucial for the development of tumors [45]. Increasing evidence suggests that the inflammatory components of the TME are diversified in cancers from different tissues [46]. Macrophage infiltration is the common denominator of different cancers and is a double-edged sword with dual potential in response to microenvironment [47, 48]. Particularly, facilitating M1 macrophage polarization is important for existing checkpoint blockade immunotherapy by inhibiting PDL1 and PDL2 [49, 50]. Previous studies have demonstrated PRNP regulates macrophage phagocytic activity, and contribute to maintaining the immunological environment [51]. Similarly, our research revealed that PRNP expression was strongly associated with M1 macrophages. Additionally, there is a strong correlation between PRNP and chemokines, immune stimulators, immune inhibitors, and receptors. Thus, a better understanding of PNRP to comprehend the heterogeneity of the tumor microenvironment, and its linkage to BRCA immunotherapy may facilitate further research.

## Conclusion

In conclusion, our study elucidated that PRNP was significantly down-regulated and showed a better prognosis value in BRCA. PRNP overexpression could enhance the inhibitory effect of gefitinib and result in increased ROS production after gefitinib treatment. Moreover, the ferroptosis selective inhibitor, ferrostatin-1, attenuated the enhancement in cellular ROS production. Immune infiltration analysis revealed PRNP correlated with macrophages, especially M1-like macrophages. Therefore, our study suggested that the Cu homeostasis-associated gene, PRNP, might be a potential target in BRCA therapy and likely functions through the regulation of ferroptosis and immune infiltration.

## Supporting information

S1 Fig Aberrant expression of PRNP in pan-cancer. **(A)** mRNA levels of PRNP based on the TIMER2 database. **(B)** Total protein level of PRNP in normal tissue and BRCA, colon cancer, ovarian cancer, clear cell RCC, UCEC, lung cancer, PAAD, head and neck, glioblastoma and liver cancer tissues from CPTAC.
(TIF)

S1 Raw images (RAR)

## References

[1] Cui G, Wu J, Lin J, Liu W, Chen P, Yu M, et al. Graphene-based nanomaterials for breast cancer treatment: promising therapeutic strategies. J Nanobiotechnology. 2021;19(1):211. Epub 2021/07/17. doi: 10.1186/s12951-021-00902-8 ; PubMed Central PMCID: PMC8281664. 34266419 PMC8281664

[2] Aghamiri S, Zandsalimi F, Raee P, Abdollahifar MA, Tan SC, Low TY, et al. Antimicrobial peptides as potential therapeutics for breast cancer. Pharmacol Res. 2021;171:105777. Epub 2021/07/24. doi: 10.1016/j.phrs.2021.105777 . 34298112

[3] Sokolova A, Johnstone KJ, McCart Reed AE, Simpson PT, Lakhani SR. Hereditary breast cancer: syndromes, tumour pathology and molecular testing. Histopathology. 2023;82(1):70–82. Epub 2022/12/06. doi: 10.1111/his.14808 . 36468211 PMC10953374

[4] Chen W, Li Z, Cheng W, Wu T, Li J, Li X, et al. Surface plasmon resonance biosensor for exosome detection based on reformative tyramine signal amplification activated by molecular aptamer beacon. J Nanobiotechnology. 2021;19(1):450. Epub 2021/12/26. doi: 10.1186/s12951-021-01210-x ; PubMed Central PMCID: PMC8709980. 34952586 PMC8709980

[5] Dreyer CA, VanderVorst K, Free S, Rowson-Hodel A, Carraway KL. The role of membrane mucin MUC4 in breast cancer metastasis. Endocr Relat Cancer. 2021;29(1):R17–R32. Epub 2021/11/03. doi: 10.1530/ERC-21-0083 ; PubMed Central PMCID: PMC8697635. 34726614 PMC8697635

[6] Collignon J, Lousberg L, Schroeder H, Jerusalem G. Triple-negative breast cancer: treatment challenges and solutions. Breast Cancer (Dove Med Press). 2016;8:93–107. Epub 2016/06/11. doi: 10.2147/BCTT.S69488 ; PubMed Central PMCID: PMC4881925. 27284266 PMC4881925

[7] Taurin S, Rosengren RJ. Raloxifene potentiates the effect of gefitinib in triple-negative breast cancer cell lines. Med Oncol. 2022;40(1):45. Epub 2022/12/10. doi: 10.1007/s12032-022-01909-3 . 36494506

[8] Fukai T, Ushio-Fukai M, Kaplan JH. Copper transporters and copper chaperones: roles in cardiovascular physiology and disease. Am J Physiol Cell Physiol. 2018;315(2):C186–C201. Epub 2018/06/07. doi: 10.1152/ajpcell.00132.2018 ; PubMed Central PMCID: PMC6139499. 29874110 PMC6139499

[9] Gupte A, Mumper RJ. Elevated copper and oxidative stress in cancer cells as a target for cancer treatment. Cancer Treat Rev. 2009;35(1):32–46. Epub 2008/09/09. doi: 10.1016/j.ctrv.2008.07.004 . 18774652

[10] Capriotti G, Piccardo A, Giovannelli E, Signore A. Targeting Copper in Cancer Imaging and Therapy: A New Theragnostic Agent. J Clin Med. 2022;12(1). Epub 2023/01/09. doi: 10.3390/jcm12010223 ; PubMed Central PMCID: PMC9821557. 36615024 PMC9821557

[11] Zeng R, Peng B, Peng E. Downregulated Copper Homeostasis-Related Gene FOXO1 as a Novel Indicator for the Prognosis and Immune Response of Breast Cancer. J Immunol Res. 2022;2022:9140461. Epub 2022/07/09. doi: 10.1155/2022/9140461 ; PubMed Central PMCID: PMC9256448 this article. 35800988 PMC9256448

[12] Hancock JL, Kalimutho M, Straube J, Lim M, Gresshoff I, Saunus JM, et al. COMMD3 loss drives invasive breast cancer growth by modulating copper homeostasis. J Exp Clin Cancer Res. 2023;42(1):90. Epub 2023/04/19. doi: 10.1186/s13046-023-02663-8 ; PubMed Central PMCID: PMC10111822. 37072858 PMC10111822

[13] Prusiner SB. Shattuck lecture—neurodegenerative diseases and prions. N Engl J Med. 2001;344(20):1516–26. Epub 2001/05/18. doi: 10.1056/NEJM200105173442006 . 11357156

[14] Choi M, Moon S, Eom HJ, Lim SM, Kim YH, Nam S. High Expression of PRNP Predicts Poor Prognosis in Korean Patients with Gastric Cancer. Cancers (Basel). 2022;14(13). Epub 2022/07/10. doi: 10.3390/cancers14133173 ; PubMed Central PMCID: PMC9264980. 35804944 PMC9264980

[15] Ryskalin L, Busceti CL, Biagioni F, Limanaqi F, Familiari P, Frati A, et al. Prion Protein in Glioblastoma Multiforme. Int J Mol Sci. 2019;20(20). Epub 2019/10/18. doi: 10.3390/ijms20205107 ; PubMed Central PMCID: PMC6834196. 31618844 PMC6834196

[16] Yousaf S, Ahmad M, Wu S, Zia MA, Ahmed I, Iqbal HMN, et al. Cellular Prion Protein Role in Cancer Biology: Is It A Potential Therapeutic Target? Biomedicines. 2022;10(11). Epub 2022/11/12. doi: 10.3390/biomedicines10112833 ; PubMed Central PMCID: PMC9687521. 36359353 PMC9687521

[17] Ding M, Chen Y, Lang Y, Cui L. The Role of Cellular Prion Protein in Cancer Biology: A Potential Therapeutic Target. Front Oncol. 2021;11:742949. Epub 2021/10/02. doi: 10.3389/fonc.2021.742949 ; PubMed Central PMCID: PMC8476782. 34595121 PMC8476782

[18] Santos EM, Fraga CAC, Xavier A, Xavier MAS, Souza MG, Jesus SF, et al. Prion protein is associated with a worse prognosis of head and neck squamous cell carcinoma. J Oral Pathol Med. 2021;50(10):985–94. Epub 2021/04/26. doi: 10.1111/jop.13188 . 33896033

[19] Muras AG, Hajj GN, Ribeiro KB, Nomizo R, Nonogaki S, Chammas R, et al. Prion protein ablation increases cellular aggregation and embolization contributing to mechanisms of metastasis. Int J Cancer. 2009;125(7):1523–31. Epub 2009/05/16. doi: 10.1002/ijc.24425 . 19444918

[20] Dery MA, Jodoin J, Ursini-Siegel J, Aleynikova O, Ferrario C, Hassan S, et al. Endoplasmic reticulum stress induces PRNP prion protein gene expression in breast cancer. Breast Cancer Res. 2013;15(2):R22. Epub 2013/03/19. doi: 10.1186/bcr3398 ; PubMed Central PMCID: PMC3672785. 23497519 PMC3672785

[21] Kretschmer C, Sterner-Kock A, Siedentopf F, Schoenegg W, Schlag PM, Kemmner W. Identification of early molecular markers for breast cancer. Mol Cancer. 2011;10(1):15. Epub 2011/02/15. doi: 10.1186/1476-4598-10-15 ; PubMed Central PMCID: PMC3045364. 21314937 PMC3045364

[22] Harvell DM, Kim J, O’Brien J, Tan AC, Borges VF, Schedin P, et al. Genomic signatures of pregnancy-associated breast cancer epithelia and stroma and their regulation by estrogens and progesterone. Horm Cancer. 2013;4(3):140–53. Epub 2013/03/13. doi: 10.1007/s12672-013-0136-z ; PubMed Central PMCID: PMC3810166. 23479404 PMC3810166

[23] Uhlen M, Zhang C, Lee S, Sjostedt E, Fagerberg L, Bidkhori G, et al. A pathology atlas of the human cancer transcriptome. Science. 2017;357(6352). Epub 2017/08/19. doi: 10.1126/science.aan2507 . 28818916

[24] Bartha A, Gyorffy B. TNMplot.com: A Web Tool for the Comparison of Gene Expression in Normal, Tumor and Metastatic Tissues. Int J Mol Sci. 2021;22(5). Epub 2021/04/04. doi: 10.3390/ijms22052622 ; PubMed Central PMCID: PMC7961455. 33807717 PMC7961455

[25] Tang Z, Kang B, Li C, Chen T, Zhang Z. GEPIA2: an enhanced web server for large-scale expression profiling and interactive analysis. Nucleic Acids Res. 2019;47(W1):W556–W60. Epub 2019/05/23. doi: 10.1093/nar/gkz430 ; PubMed Central PMCID: PMC6602440. 31114875 PMC6602440

[26] Gyorffy B. Survival analysis across the entire transcriptome identifies biomarkers with the highest prognostic power in breast cancer. Comput Struct Biotechnol J. 2021;19:4101–9. Epub 2021/09/17. doi: 10.1016/j.csbj.2021.07.014 ; PubMed Central PMCID: PMC8339292. 34527184 PMC8339292

[27] Szklarczyk D, Gable AL, Lyon D, Junge A, Wyder S, Huerta-Cepas J, et al. STRING v11: protein-protein association networks with increased coverage, supporting functional discovery in genome-wide experimental datasets. Nucleic Acids Res. 2019;47(D1):D607–D13. Epub 2018/11/27. doi: 10.1093/nar/gky1131 ; PubMed Central PMCID: PMC6323986. 30476243 PMC6323986

[28] Li T, Fu J, Zeng Z, Cohen D, Li J, Chen Q, et al. TIMER2.0 for analysis of tumor-infiltrating immune cells. Nucleic Acids Res. 2020;48(W1):W509–W14. Epub 2020/05/23. doi: 10.1093/nar/gkaa407 ; PubMed Central PMCID: PMC7319575. 32442275 PMC7319575

[29] Vasaikar SV, Straub P, Wang J, Zhang B. LinkedOmics: analyzing multi-omics data within and across 32 cancer types. Nucleic Acids Res. 2018;46(D1):D956–D63. Epub 2017/11/15. doi: 10.1093/nar/gkx1090 ; PubMed Central PMCID: PMC5753188. 29136207 PMC5753188

[30] Jiang X, Jiang Z, Xiang L, Chen X, Wu J, Jiang Z. Identification of a two-gene prognostic model associated with cytolytic activity for colon cancer. Cancer Cell Int. 2021;21(1):95. Epub 2021/02/10. doi: 10.1186/s12935-021-01782-6 ; PubMed Central PMCID: PMC7869500. 33557848 PMC7869500

[31] Ru B, Wong CN, Tong Y, Zhong JY, Zhong SSW, Wu WC, et al. TISIDB: an integrated repository portal for tumor-immune system interactions. Bioinformatics. 2019;35(20):4200–2. Epub 2019/03/25. doi: 10.1093/bioinformatics/btz210 . 30903160

[32] Han Y, Wang Y, Dong X, Sun D, Liu Z, Yue J, et al. TISCH2: expanded datasets and new tools for single-cell transcriptome analyses of the tumor microenvironment. Nucleic Acids Res. 2023;51(D1):D1425–D31. Epub 2022/11/03. doi: 10.1093/nar/gkac959 ; PubMed Central PMCID: PMC9825603. 36321662 PMC9825603

[33] Xu Z, Chen X, Song L, Yuan F, Yan Y. Matrix Remodeling-Associated Protein 8 as a Novel Indicator Contributing to Glioma Immune Response by Regulating Ferroptosis. Front Immunol. 2022;13:834595. Epub 2022/03/15. doi: 10.3389/fimmu.2022.834595 ; PubMed Central PMCID: PMC8911537. 35281049 PMC8911537

[34] Song X, Wang X, Liu Z, Yu Z. Role of GPX4-Mediated Ferroptosis in the Sensitivity of Triple Negative Breast Cancer Cells to Gefitinib. Front Oncol. 2020;10:597434. Epub 2021/01/12. doi: 10.3389/fonc.2020.597434 ; PubMed Central PMCID: PMC7785974. 33425751 PMC7785974

[35] Peng Z, Yuan L, XuHong J, Tian H, Zhang Y, Deng J, et al. Chiral nanomaterials for tumor therapy: autophagy, apoptosis, and photothermal ablation. J Nanobiotechnology. 2021;19(1):220. Epub 2021/07/24. doi: 10.1186/s12951-021-00965-7 ; PubMed Central PMCID: PMC8299636. 34294083 PMC8299636

[36] He X, Fan X, Bai B, Lu N, Zhang S, Zhang L. Pyroptosis is a critical immune-inflammatory response involved in atherosclerosis. Pharmacol Res. 2021;165:105447. Epub 2021/02/01. doi: 10.1016/j.phrs.2021.105447 . 33516832

[37] Cheng F, Peng G, Lu Y, Wang K, Ju Q, Ju Y, et al. Relationship between copper and immunity: The potential role of copper in tumor immunity. Front Oncol. 2022;12:1019153. Epub 2022/11/25. doi: 10.3389/fonc.2022.1019153 ; PubMed Central PMCID: PMC9676660. 36419894 PMC9676660

[38] Krammer C, Suhre MH, Kremmer E, Diemer C, Hess S, Schatzl HM, et al. Prion protein/protein interactions: fusion with yeast Sup35p-NM modulates cytosolic PrP aggregation in mammalian cells. FASEB J. 2008;22(3):762–73. Epub 2007/10/12. doi: 10.1096/fj.07-8733com . 17928365

[39] Bobinski R, Dutka M, Pizon M, Waksmanska W, Pielesz A. Ferroptosis, Acyl Starvation, and Breast Cancer. Mol Pharmacol. 2023;103(3):132–44. Epub 2023/02/08. doi: 10.1124/molpharm.122.000607 . 36750321

[40] Cheng Y, Song Y, Chen H, Li Q, Gao Y, Lu G, et al. Ferroptosis Mediated by Lipid Reactive Oxygen Species: A Possible Causal Link of Neuroinflammation to Neurological Disorders. Oxid Med Cell Longev. 2021;2021:5005136. Epub 2021/11/03. doi: 10.1155/2021/5005136 ; PubMed Central PMCID: PMC8557075 publication of this paper. 34725564 PMC8557075

[41] Jiang X, Stockwell BR, Conrad M. Ferroptosis: mechanisms, biology and role in disease. Nat Rev Mol Cell Biol. 2021;22(4):266–82. Epub 2021/01/27. doi: 10.1038/s41580-020-00324-8 ; PubMed Central PMCID: PMC8142022. 33495651 PMC8142022

[42] Pizon M, Schott D, Pachmann U, Schobert R, Pizon M, Wozniak M, et al. Chick Chorioallantoic Membrane (CAM) Assays as a Model of Patient-Derived Xenografts from Circulating Cancer Stem Cells (cCSCs) in Breast Cancer Patients. Cancers (Basel). 2022;14(6). Epub 2022/03/26. doi: 10.3390/cancers14061476 ; PubMed Central PMCID: PMC8946779. 35326627 PMC8946779

[43] Shen Y, Li D, Liang Q, Yang M, Pan Y, Li H. Cross-talk between cuproptosis and ferroptosis regulators defines the tumor microenvironment for the prediction of prognosis and therapies in lung adenocarcinoma. Front Immunol. 2022;13:1029092. Epub 2023/02/04. doi: 10.3389/fimmu.2022.1029092 ; PubMed Central PMCID: PMC9887127. 36733399 PMC9887127

[44] Moujaber T, Balleine RL, Gao B, Madsen I, Harnett PR, DeFazio A. New therapeutic opportunities for women with low-grade serous ovarian cancer. Endocr Relat Cancer. 2021;29(1):R1–R16. Epub 2021/10/13. doi: 10.1530/ERC-21-0191 . 34636747

[45] Mao X, Xu J, Wang W, Liang C, Hua J, Liu J, et al. Crosstalk between cancer-associated fibroblasts and immune cells in the tumor microenvironment: new findings and future perspectives. Mol Cancer. 2021;20(1):131. Epub 2021/10/13. doi: 10.1186/s12943-021-01428-1 ; PubMed Central PMCID: PMC8504100. 34635121 PMC8504100

[46] Cassetta L, Fragkogianni S, Sims AH, Swierczak A, Forrester LM, Zhang H, et al. Human Tumor-Associated Macrophage and Monocyte Transcriptional Landscapes Reveal Cancer-Specific Reprogramming, Biomarkers, and Therapeutic Targets. Cancer Cell. 2019;35(4):588–602 e10. Epub 2019/04/02. doi: 10.1016/j.ccell.2019.02.009 ; PubMed Central PMCID: PMC6472943. 30930117 PMC6472943

[47] Mantovani A, Marchesi F, Malesci A, Laghi L, Allavena P. Tumour-associated macrophages as treatment targets in oncology. Nat Rev Clin Oncol. 2017;14(7):399–416. Epub 2017/01/25. doi: 10.1038/nrclinonc.2016.217 ; PubMed Central PMCID: PMC5480600. 28117416 PMC5480600

[48] DeNardo DG, Ruffell B. Macrophages as regulators of tumour immunity and immunotherapy. Nat Rev Immunol. 2019;19(6):369–82. Epub 2019/02/06. doi: 10.1038/s41577-019-0127-6 ; PubMed Central PMCID: PMC7339861. 30718830 PMC7339861

[49] Kim IS, Gao Y, Welte T, Wang H, Liu J, Janghorban M, et al. Immuno-subtyping of breast cancer reveals distinct myeloid cell profiles and immunotherapy resistance mechanisms. Nat Cell Biol. 2019;21(9):1113–26. Epub 2019/08/28. doi: 10.1038/s41556-019-0373-7 ; PubMed Central PMCID: PMC6726554. 31451770 PMC6726554

[50] Tan Y, Sun R, Liu L, Yang D, Xiang Q, Li L, et al. Tumor suppressor DRD2 facilitates M1 macrophages and restricts NF-kappaB signaling to trigger pyroptosis in breast cancer. Theranostics. 2021;11(11):5214–31. Epub 2021/04/17. doi: 10.7150/thno.58322 ; PubMed Central PMCID: PMC8039962. 33859743 PMC8039962

[51] Nitta K, Sakudo A, Masuyama J, Xue G, Sugiura K, Onodera T. Role of cellular prion proteins in the function of macrophages and dendritic cells. Protein Pept Lett. 2009;16(3):239–46. Epub 2009/03/12. doi: 10.2174/092986609787601705 . 19275736

