# Supplementary figures and images for: Copper homeostasis-associated gene PRNP regulates ferroptosis and immune infiltration in breast cancer
Source: PLoS One. 2023 Aug 3;18(8):e0288091. doi: 10.1371/journal.pone.0288091 (PMC10399738; doi:10.1371/journal.pone.0288091)

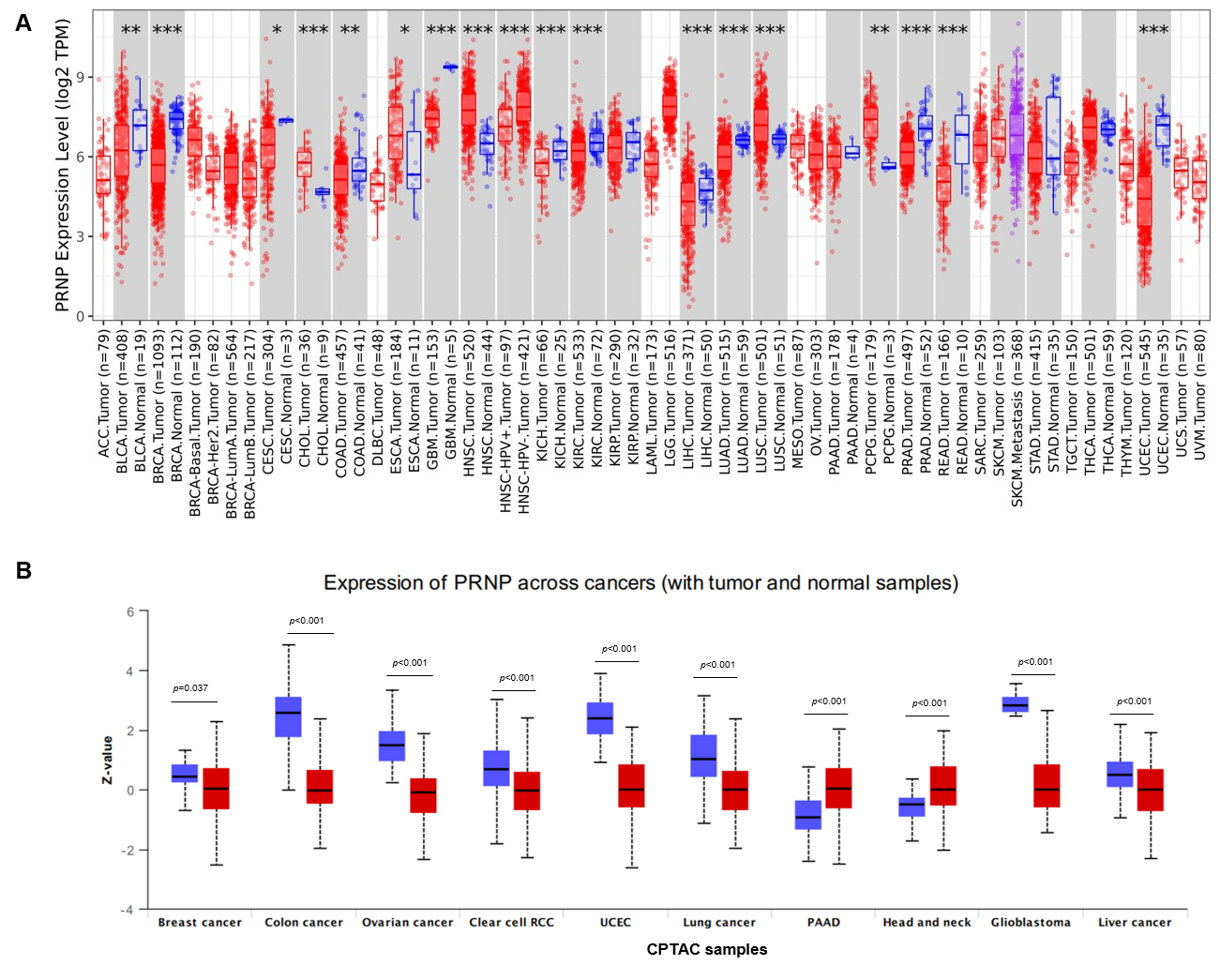

Supplement: S1 Fig — (A) mRNA levels of PRNP based on the TIMER2 database. (B) Total protein level of PRNP in normal tissue and BRCA, colon cancer, ovarian cancer, clear cell RCC, UCEC, lung cancer, PAAD, head and neck, glioblastoma and liver cancer tissues from CPTAC. (TIF) [file pone.0288091.s001.tif]
